# Minimum 7‐year Follow‐up of A Porous Coated Trabecular Titanium Cup Manufactured with Electron Beam Melting Technique in Primary Total Hip Arthroplasty

**DOI:** 10.1111/os.12846

**Published:** 2021-03-16

**Authors:** Yong Huang, Yi‐xin Zhou, Hua Tian, Jun‐wen Wang, Wen‐guang Liu, Hu Li

**Affiliations:** ^1^ Department of Orthopaedics, Beijing Jishuitan Hospital Fourth Clinical College of Peking University Beijing China; ^2^ Department of Orthopaedics and Traumatology, Wuhan Fourth Hospital (Puai Hospital) Tongji Medical College, Huazhong University of Science and Technology Wuhan China; ^3^ Orthopaedic Department Peking University Third Hospital Beijing China; ^4^ Department of Joint Surgery and Sports Medicine The Second Hospital of Shandong University Jinan China; ^5^ Department of Orthopaedic Surgery Peking University People's Hospital Beijing China

**Keywords:** 3D printing, Additive manufacturing, Electron beam melting, Primary total hip arthroplasty, Trabecular titanium cup

## Abstract

**Objectives:**

To investigate the cup survivorship, patient satisfaction level, clinical function, and radiographic outcomes of patients who underwent total hip arthroplasty (THA) using electron beam melting (EBM)‐produced porous coated titanium cups at mid‐term follow up.

**Methods:**

A total of 32 patients (32 hips) from five hospitals in China who underwent primary THA using EBM‐produced trabecular titanium cups between May and December 2012 were retrospectively reviewed. The inclusion criteria were: (i) patients who underwent THA with the use of EBM‐produced cups with possible 7‐year follow up; and (ii) patients with follow‐up information, including the cup survivorship, patient satisfaction level, and clinical outcomes such as Harris hip score. The exclusion criteria were: (i) patients with neuropathic diseases; and (ii) patients who underwent THA due to neoplastic disease. Five (15.6%) patients were lost to follow up before the 7‐year follow‐up and, thus, were excluded; none of these patients died due to disease associated with the THA or had undergone removal of their cups as of our last evaluation. The mean age and body mass index of the patients were 59.37 (range: 38.00–69.00) years and 24.51 (range: 16.50–34.10) kg/m^2^, respectively. Thirteen (48.1%) of the patients were female.

**Results:**

The average duration of follow‐up was 93.48 (range: 89.00–99.00) months. The median Harris hip score improved from 42.00 (interquartile range: 37.00–49.00) to 97.00 (interquartile range: 92.00–97.00) at the latest follow up (*P* < 0.001). A total of 18 (66.7%) patients rated their satisfaction level as very satisfied, 6 (22.2%) as satisfied, 2 (7.4%) as neutral and 1 (3.7%) as dissatisfied. No intraoperative or postoperative complications were identified. At the latest follow up, all cups were considered to have achieved osteointegration fixation, with three or more of the five signs evident in the most recent X‐ray. However, three cups revealed radiolucent lines with a width of less than 1 mm. The median vertical and horizontal distances between the latest postoperative center of rotation relative to the anatomic center of rotation were 2.50 (interquartile range: −3.10, 6.94) mm superiorly and 3.26 (interquartile range: −8.12, 2.38) mm medially, respectively, at the most recent postoperative follow up. Kaplan–Meier survivorship analysis of cups, with the endpoint defined as postoperative radiolucent lines of less than 1 mm in width in at least two zones, reveals that the 8.25‐year survival was 96.3% (95% confidence interval: 76.49%–99.47%).

**Conclusion:**

The mid‐term follow‐up of patients who underwent primary THA using EBM‐produced porous coated titanium cups demonstrated favorable patient satisfaction, good clinical function, excellent survivorship, and adequate biological fixation.

## Graphical abstract

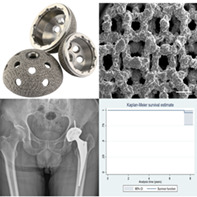

Electron beam melting (EBM) technology is an important branch of 3D printing technology and has been used in manufacturing trabecular titanium acetabular cups with high porosity (65%), diffuse pore interconnections, a low modulus of elasticity, high frictional resistance, and large holes (700 μm). The mid‐term follow up of patients who underwent primary THA using EBM‐produced porous coated titanium cups demonstrated favorable patient satisfaction, good clinical function, excellent survivorship, and adequate biological fixation.

## Introduction

While previous studies have demonstrated cemented acetabular cups' superior initial stability when compared to press‐fit acetabular cups^1^, ^2^, aseptic loosening of the cemented acetabular components results in long‐term failure of cemented primary total hip replacement as a result of their lack of secondary biologic fixation^3^, ^4^. Consequently, cementless acetabular cups were developed to achieve long‐term bone ingrowth fixation and have been the gold standard for primary total hip arthroplasty (THA). Cementless acetabular cups have achieved high survivorship in primary total hip replacement as a result of resembling the properties of trabecular bone with good biocompatibility, porosity, frictional resistance, and modulus of elasticity when compared with cemented cups. The long‐term survivorship of a cementless acetabular cup in primary THA depends on rigid primary fixation and secondary fixation *via* bone ingrowth into the voids of the porous coated surface. Threaded acetabular shells showed rigid primary stability but demonstrated a high failure rate at medium‐term to long‐term follow up due to the lack of porous coated surface and resultant inadequate osteointegration potential^1^, ^5^. Regardless of the kind of porous coated surface, the cementless acetabular components must maintain rigid initial fixation with micromotion less than 150 um, have adequate viable host bone contact, and possess an osteoconductive surface to achieve secondary biological fixation^6^, ^7^.

Since their introduction in the 1970s, cementless acetabular cups have undergone various design modifications to enhance their initial stability and long‐term biological fixation. Acetabular components with two‐dimensional surfaces for bone ongrowth, including grit‐blasted surfaces and plasma‐sprayed coated surfaces, have demonstrated favorable long‐term biological fixation *via* bone interlocking with the inherent surface irregularities on the cup^8^. Subsequently, three‐dimensional (3D) surfaces, such as sintered titanium beads and titanium fiber mesh, which rely on the ingrowth of bone into the voids of the 3D surface, have demonstrated both positive long‐term clinical outcomes and low failure rates in primary THA^9^, ^10^. Nevertheless, numerous studies have reported disadvantages of several kinds of cementless acetabular cups, including bead shedding or debonding of the porous coated surface, stress shielding, and osteolysis, which has resulted in disagreement regarding the optimal porous surface for the acetabular shells in primary THA^11^, ^12^, ^13^, ^14^.

Porous coated tantalum acetabular cups imitating trabecular bone morphology were introduced in the 1990s and have demonstrated a superior capacity for bone ingrowth as compared to HA‐coated titanium and porous coated titanium cups, especially in revision cases^15^. A novel highly‐porous coated titanium ingrowth surface known as tritanium was introduced in 2008. This highly‐porous coated titanium ingrowth surface is manufactured by depositing commercially pure titanium into an interconnected, open‐cell, polyurethane foam with a high porosity and a high coefficient of friction. However, a recent development highly porous titanium cup has demonstrated 40% radiolucent and radiosclerotic lines in two or more DeLee zones and 17.1% in all three zones in primary THA at a minimum 5‐year follow up. These cups with radiolucent and radiosclerotic lines in two or more DeLee zones were also associated with lower Harris hip scores (HHS), raising serious concerns^16^, ^17^. The fact that tritanium cups demonstrated unfavorable radiographic results in primary THA at mid‐term follow up raised severe concerns despite low revision rates^16^, ^17^ and indicated that new implant technology might not always bring positive results.

In last two decades, 3D printing technology has emerged and is being used increasingly frequently in the field of medicine due to several perceived advantages^18^. Compared with the traditional reduction casting process, 3D printing, or additive manufacturing, has made it easier to individualize product design and manufacturing. In this regard, 3D printing technology has continued to gain popularity and is becoming more widely used in preoperative education and training, preoperative planning, intraoperative cutting blocks and guide production, and for the fabrication of pelvic tumor endoprostheses in the orthopaedic arena, with favorable results. Electron beam melting (EBM) technology is an important branch of 3D printing technology and has been used in manufacturing trabecular titanium acetabular cups with high porosity (65%), diffuse pore interconnections, a low modulus of elasticity, high frictional resistance, and large holes (700 μm). This technology produces acetabular shells by building layers of porous scaffolds; in contrast, traditional reductive manufacturing produces acetabular cups by removing material from a raw shape and the porous coated surface is blasted or sprayed onto it. These EBM‐produced trabecular titanium cups are believed to induce vascularization and bone ingrowth into the interconnected pores and to reduce fibrotic peri‐acetabular tissue; these cups have demonstrated positive clinical and radiographic outcomes at midterm follow‐up^19^, ^20^, ^21^.

However, to our best knowledge, only a small number of studies exist that report the positive results of EBM‐produced trabecular titanium cups in primary THA with short‐term to mid‐term follow‐up^19^, ^20^, ^21^, ^22^, ^23^. This study aims to investigate: (i) the cup survivorship; (ii) the patient satisfaction level and clinical function; and (iii) the radiographic outcomes of patients who underwent primary THA using EBM‐produced trabecular titanium cups with mid‐term follow‐up.

## Materials and Methods

### Patients Selection

A total of 32 patients (32 hips) from five hospitals in China who underwent primary THA using EBM‐produced trabecular titanium cups between May and December 2012 were retrospectively reviewed. The 32 patients consisted of 10 patients from one hospital, 6 patients each from another two hospitals, and 5 patients each from the remaining two hospitals. The inclusion criteria were: (i) patients who underwent THA with the use of EBM‐produced cups with possible 7‐year follow up; (ii) patients with follow‐up information including the cup survivorship, patient satisfaction level, and clinical outcomes such as the HHS. The exclusion criteria were: (i) patients with neuropathic diseases; and (ii) patients who underwent THA due to neoplastic disease. Five (15.6%) patients were lost to follow up before the 7‐year follow up and, thus, were excluded from the study; none of these patients died due to disease associated with the THA or had undergone removal of their cups as of our last evaluation. Of the 27 enrolled patients, 23 had X‐rays with minimum 5‐year follow up. The average duration of follow‐up was 93.48 (range: 89.00–99.00) months. The mean age and the body mass index of the patients were 59.37 (range: 38.00–69.00) years and 24.51 (range: 16.50–34.10) kg/m^2^, respectively. Among the patients, 13 (48.1%) were female. The original diagnosis of the 27 patients included avascular necrosis in 9 (33.3%) patients, primary osteoarthritis in 11 (40.7%) patients, developmental dysplasia of the hip (DDH) in 3 (11.1%) patients, rheumatoid arthritis in 2 (7.4%) patients, and acute femoral neck fracture in 2 (7.4%) patients. The present study was commenced after approval from the institutional review board of our institution. Informed consent was obtained from all patients.

### Implants

**Fig. 1:**
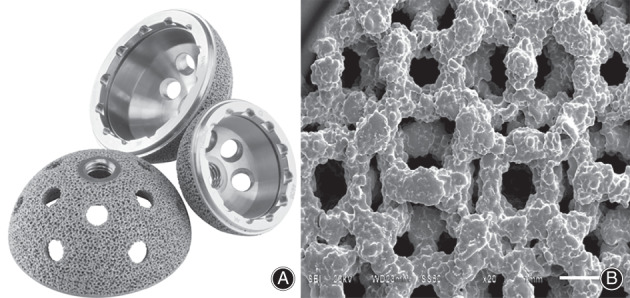
Electron beam melting (EBM)‐produced trabecular titanium acetabular cup (A) and scanning electron microscope image of the cup demonstrating interconnected trabecular titanium cellular solid structure (B).

The acetabular cup (3D ACT cup, AK Medical, Beijing, China; Fig. 1A) used in the present study was manufactured with the EBM technique^24^, ^25^. The 3D structure of the scaffold was designed using computer‐assisted design (CAD) software (Magics), and the data were stored in STL file format. The porous architecture was designed based on a dodecahedron unit cell (Fig. 1B). The cups were then prototyped using the EBM Q10 system (Acram AB, Sweden). This technique allows the melting of thin layers of metal powder, modeling a bulk construct which respects the original metal alloy properties and integrates a fine trabecular surface. This one‐step fabrication process allows structural continuity between the solid substrate part and the porous surface; thus, the EBM‐produced trabecular titanium cups possess the advantages of greater structural solidity, no risk of shedding of the coating, and a higher resistance to detachment and corrosion compared with the relative high risk of shedding with the conventional manufacturing process due to its binding interface between the porous surface and the substrate material.

The 3D ACT cups were characterized by a porosity of 50% to 80%, pore size of 600–800 μm, coefficient of friction on cancellous bone of 1.00–1.08, and modulus of elasticity of 0.5–1.3 GPa, on average. All of the pores were 100% interconnected. The diameter of the 3D ACT cup system ranges from 40 to 70 mm, with 2‐mm increments. The average cup diameter in the present study was 48.96 (range: 44–52) mm. All of the coupled bearing surfaces were ceramic on highly cross‐linked polyethylene. All of the femoral heads were BIOLOX Delta ceramic (CeramTec GmbH, Plochingen, Germany). The diameters of 24 femoral heads were 28 mm, while the other 3 head diameters were 22 mm.

### Surgery Process

All of the procedures were performed with general anesthesia or combined spinal epidural at the discretion of the anesthesiologist through a posterolateral surgical approach with the patient in the lateral decubitus position. We incised the tensor fasciae latae and split the gluteus maximus in the direction of the muscle fibers. The short extortors were cut off from the proximal femur and the hip capsular were incised. After dislocation of the femora head, the labrum, ligamentum capitis femoris, and other fat or fibrous tissue were resected, and the acetabulum was reamed until fresh bleeding bone was reached. The cups were inserted by five different consultant surgeons from five different hospitals, with 1 mm of under‐reaming to achieve a 1‐mm press fit according to the manufacturers' recommendations Additional screw fixation was used based on each surgeon's preference.

### Clinical Outcome Measures

Each patient's clinical and radiographic data were prospectively documented by the clinical staff at the time of the index procedure at each center. Patients were asked to return to the clinic for follow up at 1, 3, 6, and 12 months postoperatively and annually thereafter. The attending surgeon assessed the clinical results using the HHS^26^. All patients underwent standard anteroposterior radiographs of the bilateral hips at each follow‐up visit. Patients who were unable to return to visit the hospital postoperatively were followed up by telephone and mailed us their latest radiographs for review. Patients were asked to rate their satisfaction level of the clinical outcome on the basis of an arbitrary scale including five levels of satisfaction: very dissatisfied, dissatisfied, neutral, satisfied, or very satisfied^27^.

### Harris Hip Score

The HHS was used to assess the hip function in an adult population. The HHS mainly consists of four domains: pain, function, deformity, and range of motion. The maximum score is 100 points, which indicates the best hip function. A total score less than 70 is deemed poor, 70–80 as fair, 80–90 as good, and 90–100 as excellent.

### Radiographic outcome measures

Two independent surgeons who did not participate in the procedure and were blinded to the clinical outcomes evaluated the radiographic outcomes of the cup, including the presence of radiolucent lines in the DeLee‐Charnley zones, by reviewing all the postoperative serial radiographs. Any discrepancies between the two reviewers were discussed and the final decision was made by a third consultant senior surgeon. All of the radiographic variations in magnification were calibrated with the known head size as an internal reference.

### Cup Loosening and Osteointegration

Cup loosening was defined as the acetabular shell migrating by more than 3 mm and/or a change in inclination by more than 5° in comparison with the immediate postoperative radiographs, as well as being bordered by a progressive radiolucent line wider than 2 mm^28^, ^29^. Cup osteointegration was assessed according to the criteria of Moore *et al*.^30^, which includes the absence of a radiolucent line(s), superolateral buttress, medial stress shielding, radial trabeculae, and inferomedial buttress. A cup was considered to have osteointegrated when three or more of the five signs were visualizable on the postoperative radiographs. Cup loosening and osteointegration indicate the fixation status of the cup. Cup loosening means the cup fails and cup osteointegration means the cup achieves bone ingrowth fixation.

### Cup Inclination and Anteversion Angle

The cup inclination angle was measured with reference to the inter‐teardrop line and the anteversion angle was measured in accordance with the method of Lewinnek^31^ *et al*., both on the latest anteroposterior plain radiograph. The cup inclination and anteversion angle represent the orientation of the cup, and having a satisfactory cup inclination and anteversion angle minimizes the risk of postoperative hip dislocation.

### Vertical and Horizontal Position of the Center of Rotation

The vertical and horizontal positions of the center of rotation refer to the vertical and horizontal distances of the center of rotation to the anatomic center of the femoral head. The vertical and horizontal distances of the center of rotation to the anatomic center of the femoral head were measured on the preoperative and postoperative radiographs. A proper vertical and horizontal position of the center of rotation improves the postoperative hip biomechanics and function and increases the long‐term cup survivorship.

### Statistical Analysis

The normality of the continuous variables was examined using the Shapiro–Wilk test. Continuous data were compared using the Wilcoxon signed rank test and summarized as the median and interquartile range. Categorical data were summarized as numbers and percentages. Kaplan–Meier cup survival analysis was performed using Stata 12.0 software (Stata, College Station, TX, USA), with the endpoint defined as postoperative radiolucent lines of less than 1 mm in width in at least two zones. The significance level was set at *P* < 0.05. All statistical analyses were conducted using SPSS 17.0 for Windows (IBM, Armonk, NY, USA).

## Results

### Clinical Outcome Measures

The median HHS score improved from 42.00 (interquartile range: 37.00–49.00) to 97.00 (interquartile range: 92.00–97.00) at the latest follow up (*P* < 0.001). Eighteen (66.7%) patients rated their satisfaction level as very satisfied, 6 (22.2%) as satisfied, 2 (7.4%) as neutral, and 1 (3.7%) as dissatisfied. No patient rated themselves as very dissatisfied. The dissatisfied patient was diagnosed with rheumatoid arthritis, which had damaged multiple joints, especially the knee and ankle joints. As such, this patient had difficulty with ambulating due to hip, knee, and ankle joint pain.

### Radiographic Outcome Measures

**Fig. 2:**
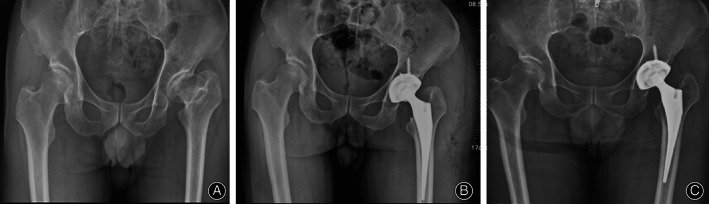
(A) The preoperative anteroposterior view of the bilateral hip joint of a 60‐year old man diagnosed as developmental dysplasia of the hip. (B) The immediate postoperative and (C) the 90‐month postoperative anteroposterior view of the bilateral hip joint demonstrating favorable bone ingrowth fixation.

**Fig. 3:**
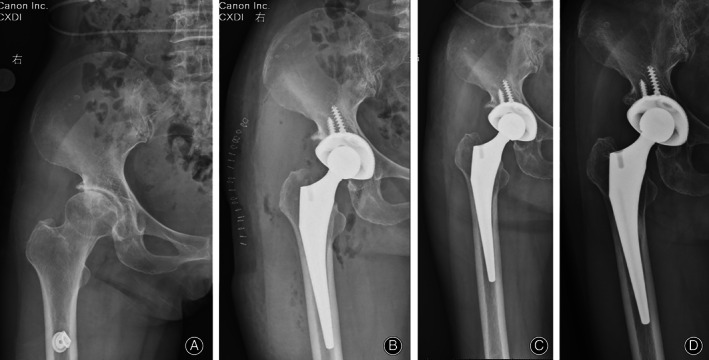
(A) The preoperative anteroposterior view of the right hip joint of a 63‐year old woman diagnosed as right femoral head necrosis. (B) The immediate postoperative, (C) the 3‐month postoperative anteroposterior view, and (D) the 60‐month postoperative anteroposterior view (D) of the right hip joint demonstrating favorable bone ingrowth fixation.

**Fig. 4:**
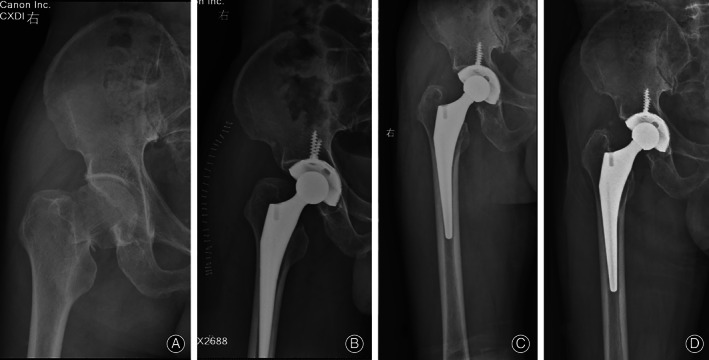
(A) The preoperative anteroposterior view of the right hip joint of a 64‐year old man diagnosed as right femoral neck fracture. (B) The immediate postoperative, (C) the 3‐month postoperative anteroposterior view, and (D) the 63‐month postoperative anteroposterior view (D) of the right hip joint demonstrating favorable bone ingrowth fixation.

At the latest follow‐up, all cups were considered to have achieved fixation *via* bone ingrowth, with three or more of the five signs identifiable on the latest radiographs (Figs 2, 3, 4). However, three cups revealed radiolucent lines of less than 1 mm in width. These radiolucent lines were distributed in DeLee–Charnley zone 1 in one patient, zone 3 in another patient, and zones 1 and 2 in the third patient.

The median vertical and horizontal distances between the latest postoperative center of rotation relative to the anatomic center of rotation were 2.50 (interquartile range: −3.10, 6.94) mm superiorly and 3.26 (interquartile range: −8.12, 2.38) mm medially, respectively, at the most recent postoperative follow up. The mean acetabular cup abduction and anteversion angles were 40.91° (interquartile range: 36.25°, 45.09°) and 16.56° (interquartile range: 13.22°, 19.84°) at the most recent postoperative follow‐up.

### Survivorship Analysis

**Fig. 5:**
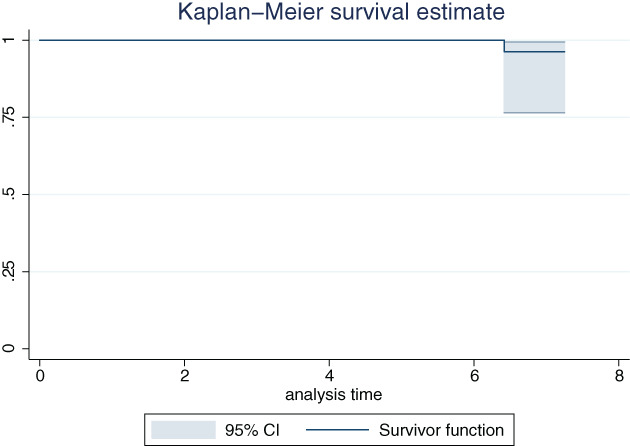
Kaplan–Meier survivorship analysis of cups with the endpoint defined as postoperative radiolucent lines of less than 1 mm in width in at least two zones. CI, confidence interval.

Kaplan–Meier survivorship analysis of cups with the endpoint defined as postoperative radiolucent lines of less than 1 mm in width in at least two zones reveals that the 8.25‐year survival was 96.3% (95% confidence interval: 76.49%–99.47%, Fig. 5).

### Complications

No intraoperative or postoperative complications were identified, including aseptic cup loosening, hip dislocation, periprosthetic joint infection, periprosthetic fracture, nerve palsy, hematoma, or incisional dehiscence.

## Discussion

Cementless acetabular cups with various surface coating modifications have demonstrated adequate biological fixation. Among these, the trabecular metal porous tatanlum surface appears noteworthy. Apart from cup surface coating modifications, the additive manufacturing techniques, specifically the EBM technique, have also been introduced in last two decades to produce trabecular titanium acetabular cups. Nevertheless, few studies have reported positive clinical and radiographic outcomes at short‐term to mid‐term follow up^19^, ^20^, ^21^, ^22^, ^23^.

### Clinical Outcome and Survivorship

Perticarini *et al*.^19^ first reported encouraging clinical and radiographic performance of the EBM‐produced trabecular titanium cups at an average follow up of 72.7 months. Castagnini^21^ *et al*. also demonstrated reliable clinical and radiographic results for trabecular titanium cups produced using the EBM technique. In another registry study^20^ enrolling 36,787 cementless cups, Castagnini *et al*. reported that EBM‐produced trabecular titanium cups (9864 patients) achieved significantly higher survival rates (98.7% *vs* 97.9%) than other cementless sockets (26,923 patients) at 7‐year follow‐up. All of the patients using the EBM‐produced trabecular titanium cups in the present study also achieved good clinical function and satisfaction levels at mid‐term follow‐up, indicating considerable improvement in pain relief, functional recovery, and quality of life. The results were consistent with and further confirmed previous findings with regards to the EBM‐produced trabecular titanium cups^19^, ^20^, ^21^, ^22^, ^23^.

The encouraging clinical outcomes and survivorship rates of the EBM‐produced trabecular titanium cups in the current study rely on the solid initial fixation and excellent osteointegration capacity. The rigid primary stability of the cup may contribute to its relatively larger coefficient of friction and low modulus of elasticity. Previous studies have demonstrated that the stability of the acetabular components is markedly influenced by the coefficient of friction^32^. Increasing the coefficient of friction may reduce interface micromotion, enhance primary fixation, and contribute to secondary fixation *via* bone ingrowth given that micromotion beyond 150 μm is known to induce a pattern of fibrous tissue ingrowth pattern at the bone–implant interface^6^. The coefficient of friction of the EBM‐produced trabecular titanium material on cancellous bone is 1.00–1.08, higher than that of trabecular metal porous tantalum on cancellous (0.98) and sintered beads on cancellous bone (0.5). The microspikes on the surface of EBM‐produced trabecular titanium cups may also facilitate its initial fixation^21^. The modulus of elasticity of the EBM‐produced trabecular titanium is 0.5–1.3 GPa, on average, which is lower than that of tantalum trabecular metal material (3 GPa). Furthermore, a low modulus of elasticity facilitates physiological stress transfer, which helps reduce the risk of stress shielding and bone resorption, as Massari^23^ *et al*. indicated in their study using the method of dual energy X‐ray absorptiometry (DEXA).

### Radiographic Outcome Measures

The fact that all the EBM‐produced highly porous trabecular titanium cups demonstrated three or more of the five signs that Moore *et al*.^30^ have proposed reflects excellent osteointergration capacity. The EBM‐produced trabecular titanium cups have a porosity of 80%, a pore size of 600–800 μm, and 100% interconnected pores. High porosity has been demonstrated to improve local vascularization, to decrease peri‐implant fibrotic tissue, and to stimulate favorable bone ingrowth^33^, ^34^. Although Taniguchi *et al*.^35^ demonstrated that the EBM‐produced pore size promotes deep bone ingrowth in an *in vivo* study, such a pore size cannot be achieved with traditional manufacturing processes. Furthermore, multiple previous studies have revealed that EBM‐produced trabecular titanium possesses good mechanical, osteoconductive, and osteoinductive properties, particularly in regard to the ability to promote osteoblast proliferation and differentiation^36^, ^37^.

Massari *et al*.^23^ observed no radiolucent lines in a 2‐year follow‐up of 91 EBM‐produced highly porous trabecular titanium cups. Perticarini *et al*.^19^ also reported no radiolucent lines around 134 EBM‐produced trabecular titanium cups at an average follow up of 72.7 months. Castagnini^21^ *et al*. reported no radiolucencies around 24 EBM‐produced trabecular titanium cups at a mean follow up of 79 months. Our study found radiolucent lines with a width of less than 1 mm in 3 patients, which is comparable to the results of the above studies. However, Imai *et al*.^22^ observed radiolucent lines in two zones in 13 DDH hips and in three zones in 2 DDH hips in a study of 101 EBM‐produced highly porous titanium cups with a mean follow‐up of 1.6 years. The authors speculated that there might be micromotion between the cups using clustered screws and the acetabular bone in cases of DDH with atrophic bone remodeling patterns. This difference may be due to the fact that patients with DDH had inadequate bone stock for cup initial fixation. Thus, Imai *et al*.^22^ suggest using multiple screws to enhance initial fixation of EBM‐produced trabecular titanium cups in such patients.

The present study has several limitations. First, this study is a case series with a small number of patients without a comparative group. Second, the patients in this study were enrolled from five different hospitals and were operated on by five different surgeons, which may confound the results; however, all cups were inserted in accordance with the manufacturer's production manual by consultant surgeons with at least 50 cases per year. Third, a radiostereometric and DEXA‐based peri‐acetabular bone quality and density evaluation was not performed on patients in the current study. Fourth, serum ion concentration measurements were not conducted to evaluate the corrosion safety of EBM‐produced cups.

## Conclusion

In summary, the mid‐term follow‐up of patients who underwent primary THA with the use of EBM‐produced porous coated titanium cups demonstrated favorable patient satisfaction levels, good clinical function, excellent survivorship, and adequate biological fixation. However, as with all new implant technology, this should be used in a stepwise process. The outcomes of the EBM‐produced trabecular titanium cups remain to be investigated in a larger volume of patients and at longer‐term follow‐up.
